# Associations of the intestinal microbiome with the complement system in neovascular age-related macular degeneration

**DOI:** 10.1038/s41525-020-00141-0

**Published:** 2020-09-01

**Authors:** Denise C. Zysset-Burri, Irene Keller, Lieselotte E. Berger, Carlo R. Largiadèr, Matthias Wittwer, Sebastian Wolf, Martin S. Zinkernagel

**Affiliations:** 1 Department of Ophthalmology, Inselspital, Bern University Hospital, University of Bern, Bern, Switzerland; 2 Department for BioMedical Research, University of Bern, Bern, Switzerland; 3 Interfaculty Bioinformatics Unit and Swiss Institute of Bioinformatics, University of Bern, Bern, Switzerland; 4 Department of Clinical Chemistry, Inselspital, Bern University Hospital, University of Bern, Bern, Switzerland; 5 Spiez Laboratory, Federal Office for Civil Protection, Austrasse, Spiez, Switzerland

**Keywords:** Macular degeneration, Metagenomics

## Abstract

Age-related macular degeneration (AMD) is a leading cause of severe vision loss in the aged population. The etiology of AMD is multifactorial including nutritional factors, genetic variants mainly in the complement pathway, environmental risk factors and alterations in the intestinal microbiome. However, it remains unexplored whether there is an interdependency of these factors leading to the development of AMD. To investigate this issue, a shotgun metagenomics analysis of 57 neovascular AMD and 58 healthy controls as well as of 16 complement C3-deficient mice and 16 wildtypes was performed. Whereas the class *Negativicutes* was more abundant in patients, the genus *Oscillibacter* and species *Bacteroides* had a significantly higher prevalence in persons without AMD. Similar taxonomic features were identified that distinguished wildtype mice from C3-deficient mice. Moreover, several purine signaling pathways were associated with both, neovascular AMD and C3 deficiency. While SNPs within the *complement factor B* gene were more abundant in controls, SNPs within the *high temperature requirement A serine peptidase 1* and *complement factor H* (CFH) genes were associated with neovascular AMD. Using a classification model, *Negativicutes* was identified as a potential biomarker for AMD and furthermore, it positively correlated with CFH. This study suggests an association between the intestinal microbiome and the complement system in neovascular AMD.

## Introduction

Age-related macular degeneration (AMD) is the most frequent cause of blindness among older people in developed countries^1^. AMD has become a significant economic and social burden on public health^1^ and its prevalence is projected to grow by 50% in the next decades^2^. The early stage of AMD results in accumulation of extracellular material underneath the retina, called drusen. Drusen are a sign of impaired retinal pigment epithelium (RPE) function and disruption of the metabolic transport between RPE and choroid^3^. Whereas the intermediate stage of AMD is characterized by larger drusen, the late stages are characterized by either choroidal neovascularization (CNV) in the exudative form or geographic atrophy in the atrophic form of AMD^4^. The pathogenesis of AMD is multifactorial and is thought to be a combination of oxidative stress, impaired RPE function, increased apoptosis, and aberrant immune system activation^5^.

Despite major research efforts, treatment options for early and intermediate AMD are only very limited. Smoking is the strongest modifiable risk factor for AMD, resulting in oxidative stress, ischemia, hypoxia, and ultimately development of CNV. Other environmental risk factors are sunlight exposure^6,7^ and obesity^8^. To date, the only factor that has been shown to be protective is a healthy diet, rich in omega-3 fatty acids, lutein, zeaxanthin, and antioxidants, consistent with the Age-Related Eye Disease Study 2 (AREDS2) formulation^9^. Therefore, preventative strategies targeting nutritional intake to avoid the development of AMD seem to be most promising^9–14^. Recently, we have shown that compositional and functional features of the intestinal microbiome may have an impact on the development and progression of AMD^15^. Moreover, in an animal model, it has been shown that modifications in the intestinal microbiome resulted in exacerbation of CNV^16^.

In addition to the effect of modifiable environmental risk factors, genetic polymorphisms in genes such as in complement factor H (CFH) may be linked with AMD^17^. Genetic polymorphisms in components of the complement system refer to the potential role of local inflammation and complement regulation in the pathogenesis of AMD. Moreover, since toll-like receptors (TLRs) recognize microbe-specific molecules and are expressed on various cells along the gastrointestinal tract, they may be crucial in signaling between the immune system and the microbiota. Moreover, the complement system is a key part of the innate immune system operating via TLR signaling. When over-activated or de-regulated, the complement becomes a major link between infection and inflammation with profound involvement in inflammatory and degenerative diseases such as AMD^18^. In this study, we investigated gut microbiome alterations in patients with neovascular AMD and screened for associations between the intestinal microbiome and the complement system.

## Results

### Taxonomic and functional characterization of the intestinal microbiome in AMD patients and healthy controls

**Fig. 1:**
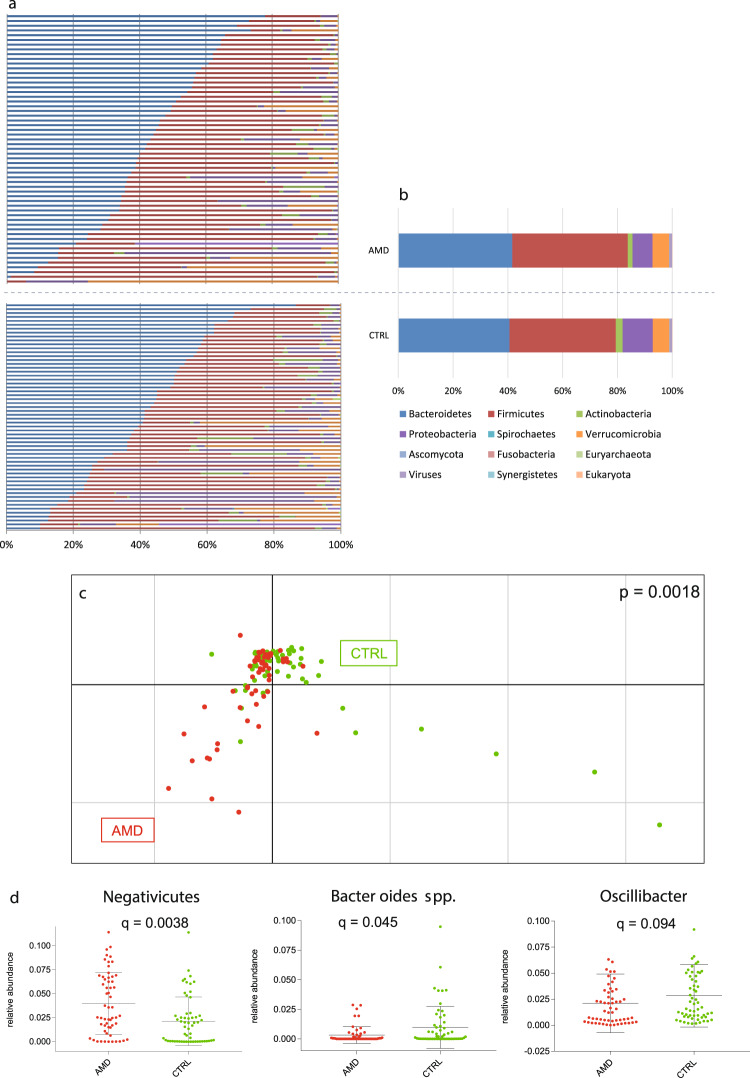
Diversity of the intestinal microbiome in AMD patients and healthy controls. Relative abundances of microbiota at phyla level in all study subjects (**a**) and averaged for study groups **(b**). **c** Principal component analysis of microbial species abundance grouped patients and controls separately, with PERMANOVA confirming a significant difference between the groups (*p* = 0.0018). **d** Relative abundances of taxa associated with AMD (*q*-values after adjusting for false discovery rate). Mean value and standard deviation are shown. *AMD* age-related macular degeneration; *CTRL* control.

In total, 115 stool samples were sequenced from 57 neovascular AMD patients and 58 age- and sex-matched healthy controls. The AMD patients and controls were similar for gender, age at collection, smoking habit and BMI (*p* > 0.05, Table 1). A total of 2.8 billion 100 bp paired-end reads with an average insert size of 350 bp were generated, with an average of 24.3 ± 9.3 (s.d.) million reads per sample. After trimming and filtering, we kept about 2.5 billion non-human high-quality reads, with an average of 21.5 ± 8.5 (s.d.) million reads per sample. We identified 834 taxa, the majority of which were bacterial with 98.8 ± 8.1% (s.d.). The phyla *Firmicutes* (40.4%) and *Bacteroidetes* (41.1%) dominated the microbiome composition (Fig. 1). *Bacteroidia* (41.1%) and *Clostridia* (37.1%) were the most abundant classes, and *Bacteroides* (16.5%), *Alistipes* (13.0%), and *Subdoligranulum* (11.9%) the most abundant genera in the cohort, consistent with previous observations^19,20^.

**Table 1:** Characteristics of study patients.

Feature	Patients (*n* = 57)	Controls (*n* = 58)	*p*-value AMD vs CTRL
Males (*n* (%))	21 (36.8)	25 (43.1)	0.57^a^
Age (years)	75.4 ± 8.3	75.3 ± 8.1	0.92^b^
Current smoker (*n* (%))	6 (10.5)	3 (5.2)	0.31^a^
Previous smoker (*n* (%))	20 (35.1)	21 (36.2)	1.0^a^
BMI (kg/m^2^)	25.0 ± 4.5	26.0 ± 3.9	0.21^b^

Data are mean ± SD.

*BMI* body mass index, *CTRL* control, *AMD* age-related macular degeneration.

^a^Fisher’s exact test.

^b^Welch’s *t*-test.

**Fig. 2:**
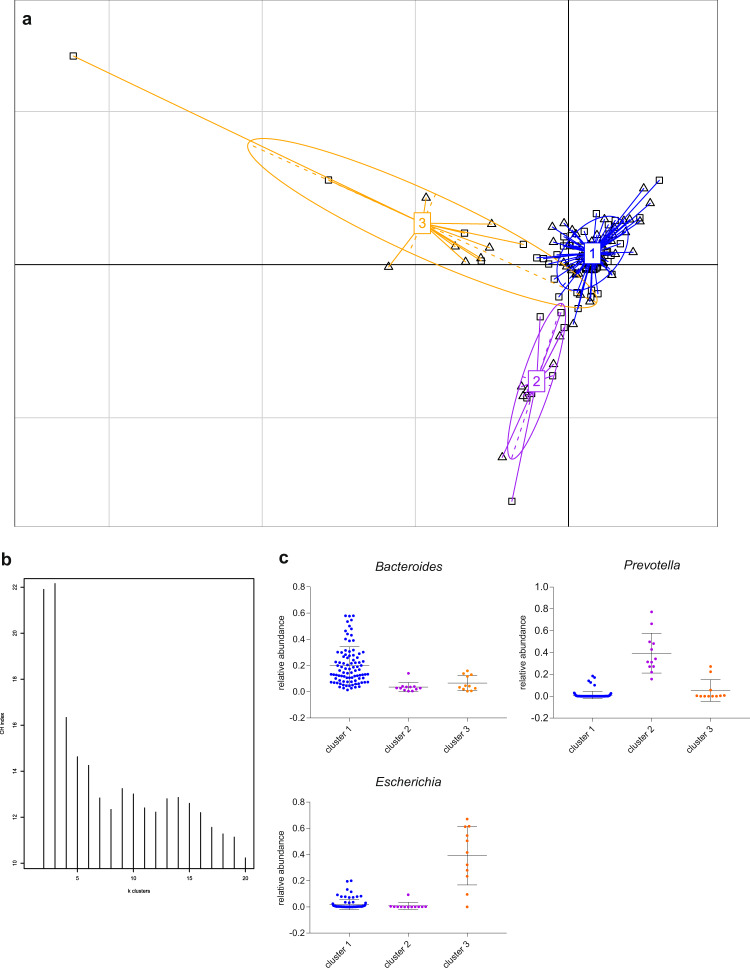
Enterotypes of the intestinal microbiome in AMD patients and healthy controls. **a** The intestinal microbiota was clustered into three enterotypes at genus level, dominated by *Bacteroides* (enterotype 1), *Prevotella* (enterotype 2), and *Escherichia* (enterotype 3). Patients (*n* = 57) and controls (*n* = 58) were denoted by squares (□) and triangles (∆), respectively. **b** The optimal number of enterotypes was three as indicated by the maximum Calinski–Harabasz (CH) index at three clusters. **c** Relative abundances of the dominant genera in the three enterotypes. Mean value and standard deviation are shown. Blue is enterotype 1, purple is enterotype 2, and orange is enterotype 3.

Arumugam et al.^19^ suggested that the human intestinal microbiome can be separated into three distinct enterotypes according to microbial composition. To cluster the AMD patients and control samples into enterotypes, we used the PAM algorithm applying Jensen–Shannon distance for relative genus abundances (Fig. 2). The optimal number of clusters was three according to the CH index (Fig. 2b). Between-class-analysis was applied to divide the samples into three enterotypes. (Fig. 2a). The following genera characterized the individual enterotypes: in enterotype 1 *Bacteroides* was present to a relatively high level, *Prevotella* in enterotype 2, and *Escherichia* was indicative for enterotype 3 (Fig. 2c). However, since no association between the enterotypes and the disease status was found (*p* > 0.05, Fisher’s exact test), the samples appear to be uniformly distributed across the three enterotypes.

**Fig. 3:**
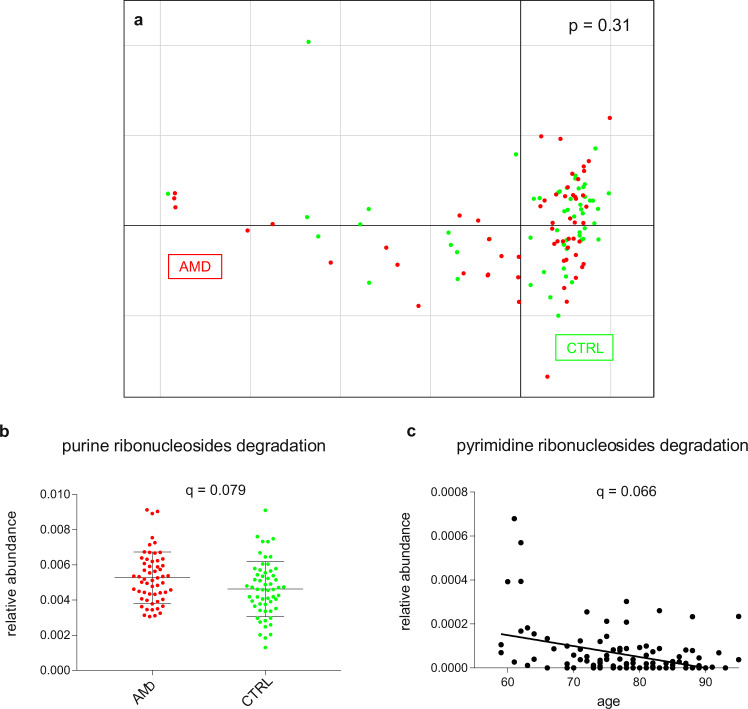
Functional profile of the intestinal microbiome in AMD patients and healthy controls. **a** Principal component analysis of functional feature abundance did not separate patients from controls (*p* = 0.31, PERMANOVA). Relative abundances of pathway associated with AMD (**b** mean value and standard deviation are shown) and age (**c** linear regression); *q*-values after adjusting for false discovery rate are shown. *AMD* age-related macular degeneration; *CTRL* control.

A principal component analysis (PCA) using the health status as grouping variable confirmed that the patient group is separated from the control group based on differences in microbial species abundances (*p* = 0.001, PERMANOVA analysis with nrepet = 10,000, Fig. 1c). However, the two groups could not be separated by differences in relative abundances of functional profiles (*p* = 0.31, PERMANOVA analysis with nrepet = 10,000, Fig. 3a). To further examine features of the intestinal microbiome in AMD patients, we compared the relative abundances of taxa between patients and controls, showing that the class *Negativicutes* was more abundant in patients (*q* = 0.0038) and the genus *Oscillibacter* (*q* = 0.094) and *Bacteroides* species (*q* = 0.045, MaAsLin) were more abundant in controls (Fig. 1d). Moreover, patient’s intestinal microbiomes were enriched in genes of the purine ribonucleosides degradation pathway (*q* = 0.079, MaAsLin, Fig. 3b).

### Identification of AMD patients based on the intestinal microbiome

**Fig. 4:**
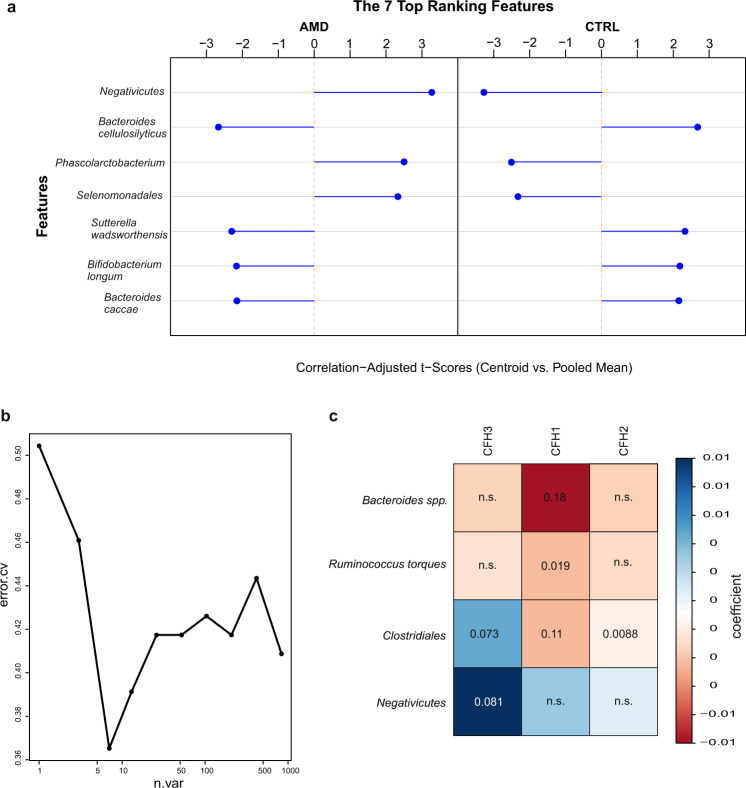
Classification to differentiate samples between AMD patients and healthy controls. **a** List of the top seven ranked biomarkers for AMD. Ranking was performed on correlation-adjusted *t* (cat) scores. The length and direction of the blue bars indicated the influence of a given biomarker on the discriminative power of the prediction model. The class *Negativicutes* had the highest potential for the separation of AMD patients and healthy controls with a positive cat score indicating an over-representation in patients. **b** Cross-validation with sequentially reduced number of predictors indicated that the prediction error was minimized at seven features. **c** Correlation between microbial taxa and clinical metadata. *R*-coefficients and corresponding *q*-values from MaAsLin are shown. Coefficients are indicated using a color gradient. Red indicates negative correlation, blue positive correlation. *AMD* age-related macular degeneration; *CTRL* control.

To illustrate the potential diagnostic value of the intestinal microbiome for AMD, a species-based classifier was used in an attempt to predict AMD samples among a mixture of samples from patients and healthy controls. Based on the relative species abundance profile from MetaPhlAn, a classification model was constructed using linear shrinkage discriminant analysis and features were ranked according to their correlation-adjusted *t* (cat) scores. A graphical visualization of the cat scores is provided in Fig. 4a. The blue bars in this figure indicate the ability of a potential biomarker to discriminate between AMD patients and controls in the prediction model. A positive cat score implies that the taxon is over-represented in AMD, whereas a negative score represents an over-represented taxon in controls. The minimum error was reached with seven features (Fig. 4b), which were selected as potential biomarkers, including the class *Negativicutes*, the order *Selenomonadales* and the species *Phascolarctobacterium, Bacteroides cellulosilyticus, Sutterella wadsworthensis, Bifidobacterium longum*, and *Bacteroides caccae*.

### Association between single nucleotide polymorphisms and AMD

Out of the genetic polymorphisms previously shown to be associated with risk for AMD^21–34^, single nucleotide polymorphisms rs800292 (*p* = 0.0012), rs1410996 (*p* = 0.00019) and rs1061170 (*p* = 0.000064) within the *complement factor H (CFH)* gene and rs11200638 (*p* = 0.00012) within the *high temperature requirement A serine peptidase 1 (HTRA1)* gene were associated with a higher risk for having AMD in our cohort. However, rs12614 (*p* = 0.0057) within the *complement factor B (CFB)* gene was inversely correlated with AMD in the cohort (Fisher’s exact test, Table 2).

**Table 2:** Single nucleotide polymorphisms in AMD.

Genes	SNP	A1	A2	F_A1	F_U	*p*-value	OR	95% CI	FDR BH
*C2*	rs1883025	C	G	0.018	0.052	0.28	0.34	(0.067, 1.72)	0.31
*C3*	rs4420638	C	G	0.25	0.16	0.10	1.74	(0.91, 3.35)	0.25
*CETP*	rs10490924	A	C	0.34	0.35	0.89	0.93	(0.54, 1.61)	0.85
*CFB1*	rs4151667	A	T	0.027	0.052	0.50	0.51	(0.13, 2.11)	0.51
*CFB2*	rs641153	T	C	0.12	0.094	0.67	1.28	(0.55, 2.99)	0.76
*CFB3*	rs12614	A	G	0.027	0.13	0.0057	0.19	(0.053, 0.67)	0.015
*CFH1*	rs800292	A	G	0.24	0.082	0.0012	3.45	(2.31, 5.63)	0.0048
*CFH2*	rs1410996	A	G	0.39	0.16	0.00019	3.21	(2.16, 4.85)	0.00091
*CFH3*	rs1061170	T	C	0.60	0.34	0.000064	3.03	(1.89, 4.57)	0.00085
*CFI*	rs10033900	C	T	0.55	0.45	0.18	1.48	(0.87, 2.50)	0.31
*COL8A1*	rs13095226	C	T	0.13	0.11	0.83	1.16	(0.52, 2.58)	0.85
*HTRA1*	rs11200638	A	G	0.51	0.26	0.00012	2.97	(1.70, 5.20)	0.00085
*LIPC*	rs10468017	T	C	0.28	0.29	0.88	0.95	(0.53, 1.68)	0.85
*TIMP3*	rs9621532	C	A	0.0091	0.034	0.37	0.26	(0.028, 2.34)	0.31
*TNFRSF10A*	rs13278062	C	A	0.41	0.5	0.18	0.69	(0.41, 1.17)	0.31
*VEGF A*	rs1413711	T	C	0.47	0.46	0.89	1.07	(0.63, 1.80)	0.85

*A1* minor allele, *A2* major allele, *F_A1* frequency of A1 in AMD, *F_U* frequency of A1 in controls, *FDR BH* false discovery rate correction using the Benjamini–Hochberg procedure, *OR* odds ratio for A1.

### Association between the intestinal microbiome and clinical metadata in the cohort

Next, we use multivariate association by linear models (MaAsLin) to examine whether relative abundances of microbial taxa and pathways were associated with demographic parameters or with genetic risk factors for AMD (*CFH1, CFH2, CFH3,* and *HTRA1*, Fig. 4c). A boosting step in the MaAsLin algorithm ensures that only variables that are associated with the given taxon are included in the linear model, implying that all associations found by the modeling approach have been corrected for all other confounding factors. For demographic parameters, age at collection positively correlated with the species *Bacteroides uniformis*, *Odoribacter unclassified,* and *Eubacterium eligens* and negatively correlated with the superpathway of pyrimidine ribonucleosides degradation (Fig. 3c; *q* < 0.2). There was no association of sex, smoking habit, or BMI with the intestinal microbiome in our cohort (*q* > 0.2). *Bacteroides species* and *Ruminococcus torques* negatively correlated with *CFH1* variants, i.e., *Bacteroides species* and *Ruminococcus torques* were less abundant in individuals possessing the SNP in the *CFH1* gene. The order *Clostridiales* positively correlated with *CFH3* and negatively correlated with *CFH1* and *CFH2* variants. The class *Negativicutes* positively correlated with CFH3 (Fig. 4C). There was no association of *HTRA1* variants with the intestinal microbiome in the cohort.

### Taxonomic and functional characterization of the intestinal microbiome in C3-deficient mice and wildtypes

**Fig. 5:**
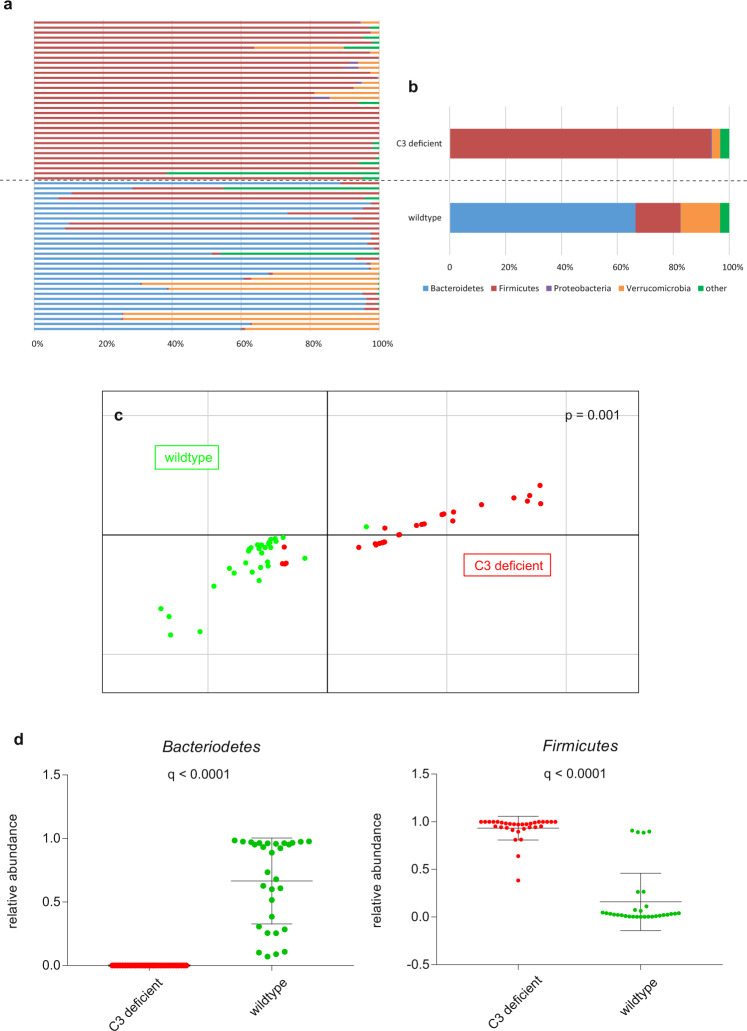
Diversity of the intestinal microbiome in C3-deficient mice and wildtypes. Relative abundances of microbiota at phyla level in all mice (**a**) and averaged for groups (**b**). **c** Principal component analysis of microbial species abundance grouped C3-deficient mice and wildtypes separately, with PERMANOVA confirming a significant difference between the groups (*p* = 0.001). Representative data of one experiment is shown. **d** Relative abundances of taxa associated with C3 deficiency (*q*-values after adjusting for false discovery rate). Mean value and standard deviation are shown.

In total, 64 stool samples were sequenced from 16 C3^−/−^ mice and 16 C57BL/6 control mice (Table 3). A total of 1.9 billion 100 bp paired-end reads with an average insert size of 350 bp were generated, with an average of 31.2 ± 11.1 (s.d.) million reads per sample. After trimming and filtering, we kept about 1.7 billion non-murine high-quality reads, with an average of 27.3 ± 9.9 (s.d.) million reads per sample. We identified 98 taxa, the majority of which were bacterial with 96.8 ± 11.1% (s.d.). The phylum *Firmicutes* or *Bacteroidetes* dominated the microbiome composition (Fig. 5a) in accordance with previous observations^35^.

**Table 3:** Parameters of animal experiments.

Group	Mouse	Timepoints (weeks)	Sex (m/w)	Number of mice
1	B6;129S4-C3^tm1Crr^/J	1 + 4	m	8
2	B6;129S4-C3^tm1Crr^/J	1 + 4	f	8
3	C57BL/6J	1 + 4	m	8
4	C57BL/6J	1 + 4	f	8 (6)

Two wildtype males were killed due to fighting activities before stool sampling have been performed at week 4.

*B6;129S4-C3tm1Crr/J* complement factor C3-deficient mice, *C57BL/6J* wildtypes.

**Fig. 6:**
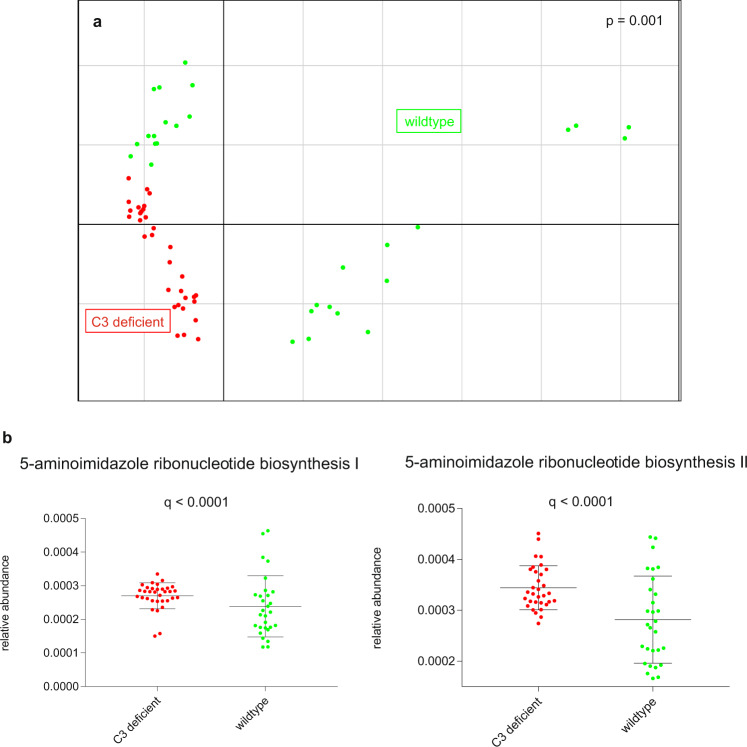
Functional profile of the intestinal microbiome in C3-deficient mice and wildtypes. **a** Principal component analysis of functional feature abundance grouped C3-deficient mice and wildtypes separately, with PERMANOVA confirming a significant difference between the groups (*p* = 0.001). **b** Relative abundances of pathway associated with C3 deficiency (*q*-values after adjusting for false discovery rate). Mean value and standard deviation are shown.

A PCA showed that differences in microbial species and functional feature abundances separated the C3^−/−^ mice from the wildtypes (*p* = 0.001, PERMANOVA analysis with nrepet = 10,000, Figs. 5c and 6a). To further examine features of the intestinal microbiome in C3^−/−^ mice, we compared the relative abundances of taxa between C3-deficient mice and wildtypes, showing that the phylum *Firmicutes* was more abundant in C3^−/−^ mice (*q* < 0.2) and the phylum *Bacteroidetes* was more abundant in wildtypes (*q* < 0.2; Fig. 5d). Based on a classification model using shrinkage discriminant analysis and ranking features according to cat scores, the phylum *Firmicutes*, the class and order *Clostridia* and *Clostridiales*, respectively, as well as the genus *Subdoligranulum* and an unclassified species of genus *Subdoligranulum* were identified as potential biomarkers for C3 deficiency in mice (Supplementary Fig. 1). Moreover, intestinal microbiomes of C3^−/−^ mice were enriched in genes of the 5-aminoimidazole ribonucleotide biosynthesis pathways (*q* < 0.0001, MaAsLin, Fig. 6b). There was no association of sex or week at collection with the intestinal microbiome in the cohort (*q* > 0.02).

## Discussion

Despite advances in the treatment of neovascular AMD, effective preventative strategies of this disease are lacking. An association of diet with development of advanced AMD has been shown in various studies (AREDS I and II). In this study, we investigated associations between the intestinal microbiome and the complement system in neovascular AMD. Microbiota in AMD patients and healthy controls was analyzed in terms of taxonomic and functional profiles and correlated to clinical metadata and genetic risk factors including SNPs in genes encoding for complement factors. We identified several taxonomic and functional features of the intestinal microbiome that differ between patients and healthy controls in their relative abundance, suggesting that they may be associated with the development of AMD. The genus *Oscillibacter* and species *Bacteroides* were more abundant in healthy controls relative to AMD patients. In contrast, the class *Negativicutes* was more abundant in AMD patients compared to controls. These results are consistent with previous studies, reporting associations between several of these taxa and AMD^15,36^. Moreover, patient’s intestinal microbiomes were enriched in genes of the purine ribonucleosides degradation pathway. Aberrant purine signaling pathways have been implicated in immune dysregulation as occurred in the pathogenesis of autoimmune diseases^37^. Although autoimmunity in terms of autoantibody production is associated with a variety of ocular diseases including AMD, the detailed triggers for ocular autoimmunity are unknown^38^. The different functional feature between patients and controls in our study suggests that immune dysregulation in AMD may occur via purine signaling.

After smoking, obesity is the second most important environmental risk factor for AMD^39^. Numerous studies have shown that a higher relative abundance of *Firmicutes* and a lower *Bacteroidetes* abundance are typically associated with obesity^40^. In a previous study, we observed a shift of relative abundance in *Firmicute*s at the expense of *Bacteroidetes* in AMD patients^15^. In this study, the class *Negativicutes* belonging to the phylum *Firmicute*s was enriched in AMD patients. Moreover, *Bacteroides* species belonging to the phylum *Bacteroidetes* was more abundant in controls. Looking at the prediction model in Fig. 4a, *Negativicutes* as well as the order *Selenomonadales*, both belonging to the phylum *Firmicutes*, may be identified as potential biomarkers for AMD. On the other hand, *Bacteroides* species, especially including *B. cellulosilyticus* and *B. caccae*, may have a protective effect on the development of AMD as these species were more abundant in the control group. Taken together, these results suggest an elevated *Firmicutes* to *Bacteroidetes* ratio, also at lower taxonomic levels in AMD patients compared to healthy controls. These data are also consistent with observations in mice, demonstrating that high-fat diets exacerbate choroidal neovascularization, which is a hallmark of exudative AMD, by increasing the relative abundance of *Firmicutes*^36^.

In addition to environmental risk factors, genetic risk factors mostly associated with the complement system are involved in the development of AMD. The first genome-wide association study performed in 2005 revealed a high association between genetic polymorphisms in complement factor H (CFH) and AMD^41^. This genetic link was further extended by several studies showing an association between AMD and other complement factors such as complement component 3 (C3), complement component C2 (C2), and complement factor B (CFB)^42,43^. In our cohort, while single nucleotide polymorphisms (SNPs) within the *CFH* gene are associated with a higher risk for developing AMD, SNP within the *CFB* gene has a protective effect (Table 2). Furthermore, to assess associations between the complement system and the intestinal microbiome, both involved into the development of AMD, microbiota of C3-deficient mice and wildtypes was analyzed in terms of taxonomic and functional profile and correlated to clinical metadata. Hoh Kam et al.^44^ showed that C3 deficiency in mice negatively affect aged retinas in terms of retinal function, inflammation and morphological features. Other publications have shown that overexpression of C3 leads to pathology in the murine retina including functional and morphological features observed in AMD^45^. However, in our study, the intestinal microbiomes of C3-deficient mice were enriched in genes of the 5-aminoimidazole ribonucleotide biosynthesis pathways. Since 5-aminoimidazole ribonucleotide is an intermediate of purine nucleotide biosynthesis and purine signaling, in turn, may be involved in AMD pathogenesis, this pathway may be a key point in the interconnection between the intestinal microbiome, the complement system and AMD. Furthermore, while the phylum *Firmicutes* was more abundant, the phylum *Bacteroidete*s was decreased in C3-deficient mice compared to wildtypes (Fig. 5). Since *Firmicutes* was proposed as potential biomarker for AMD, it may also contribute to the development of AMD in C3-deficient mice. These results show that the complement alters the intestinal microbiota composition in mice and as such microbiota-derived nutrients, metabolites and antigens within the host. This in turn may promote degenerative diseases such as AMD. This association between the complement system and the intestinal microbiome was further confirmed in humans by the correlation of SNPs within the *CFH* gene with the taxa *Negativicutes, Clostridiales, Bacteroides* species, and *Ruminococcus torques* (Fig. 4c). *Clostridiales* in particular was also identified as potential biomarker for C3 deficiency in mice, thereby highlighting the possible role of this taxon in the interconnection between complement and microbiome. However, complement factor H is the soluble inhibitor of the alternative pathway of complement, mediating anti-inflammatory housekeeping functions by protecting cells from complement activation. Thus, mutations in the *CFH* gene result in uncontrolled complement activation, which is involved in the pathogenesis of AMD^46^. Since *Negativicutes*, which was proposed as potential biomarker for AMD (Fig. 4a), positively correlated with CFH, it may influence the development and/or progression of AMD via the regulation of the alternative pathway of complement. Moreover, while the relative abundance of *Bacteroides* species were proposed as potential biomarker for controls and negatively correlated with CFH, they may be protective for AMD. Thus, further investigation could aim at modulating the intestinal microbiome of AMD patients with the aim of decreasing the *Firmicute*s to *Bacteriodetes* ratio, thereby potentially also regulate the complement pathway involved in AMD pathogenesis. However, limitations of this study include the lack of longitudinal data and the limited number of patients.

In conclusion, we found alterations of the intestinal microbiome in AMD patients. While the phylum *Firmicutes* was more abundant in patients with neovascular AMD, species *Bacteroides* may be protective for AMD, both correlating with the genetic risk factor CFH. These data point towards a possible interconnection between the intestinal microbiome, the complement system and the development of neovascular AMD. However, the data do not allow to determine whether the altered intestinal microbiome is the consequence of the disease or is involved in its pathogenesis via uncontrolled activation of the complement system.

## Methods

### Study design and recruitment

The study was approved by the Ethics Committee of the Canton of Bern (ClinicalTrials.gov: NCT02438111). Study procedures followed the tenets of the Declarations of Helsinki and the ﻿International Ethical Guidelines for Biomedical Research involving Human Subjects. Written consent was obtained from all participants before study enrollment. Participants (*n* = 115) were recruited from the Department of Ophthalmology of the University Hospital Bern (Inselspital), Switzerland. All participants were Caucasian and were 50 years of age or older. They were subjected to an ophthalmic examination including optical coherence tomography and standard fundus color photography. Patients (*n* = 57) had clinically confirmed active neovascular AMD. The control group (*n* = 58) was selected to represent an age- and sex-matched group with no signs of AMD. Participants with chronic inflammatory or gastrointestinal diseases (including previous surgery in the gastrointestinal tract) or use of systemic antibiotics within the last three months were excluded.

### Metagenomic DNA sequencing and annotation

Stool samples were provided refrigerated to the study center within 16 hours after fecal output where they were immediately frozen at −20 °C. Metagenomic DNA was isolated and sequenced as described in Zysset-Burri et al.^47^. To exclude low-quality reads and reads mapping to human DNA, the resulting 150 bp paired-end reads were quality filtered with Trimmomatic v.0.32^48^ and mapped to the human reference genome hg19 using Bowtie2 v.2.2.4^49^. For taxonomical annotation, the Metagenomic Phylogenetic Analysis tool v.2.6.0 (MetaPhlAn2) and the marker database v.20^50^ applying default settings were used. The relative abundances of each taxonomic unit were calculated using Bowtie2 v.2.2.4 for alignment followed by normalization of the total number of reads in each clade by the nucleotide length of its marker. For functional annotation, the HMP (Human Microbiome Project^51^) Unified Metabolic Analysis Network (HUMAnN2 v.0.11.0^52^) was used with default settings. In order to provide a functional interpretation of the taxonomic profiles from MetaPhlAn2, HUMAnN2 was run for each sample separately as described in Zysset-Burri et al.^47^, finally resulting in a set of pathways including their abundances.

### Genetic analysis of germline DNA

Genomic DNA was extracted from EDTA blood samples using a QIAamp DNA Blood Midi Kit (Qiagen AG, Basel, Switzerland). To identify genetic risk factors for AMD, 16 previously described SNPs were analyzed (Supplementary Table 1). Real-time quantitative PCR (see below) was used for allelic discrimination of all SNPs analyzed except for CFB (rs4151667, rs12614, and rs641153), which was assessed by Sanger sequencing. This locus was amplified using the Multiplex PCR Kit (Qiagen, Hombrechtikon, Switzerland) with an initial 15 min denaturation step at 95 °C, followed by 35 amplification cycles (30 s at 94 °C, 90 s at 58 °C, 60 s 72 °C) and a final 10 min extension at 72 °C. The following forward and reverse primers were used: 5′-GGTCTAGGTCTGGAGTTTCAGC-3′ and 5′-TTGGTCTTGAGTCTTCAGGGTG-3′. PCR products were purified using the QIAquick PCR Purification Kit (Qiagen) and Sanger sequenced using the same primers as used for PCR amplification. Bi-directional sequencing was carried out using the BigDye® Terminator v3.1 Cycle Sequencing Kit (Life Technologies, Zug, Switzerland) on a 3130xl Genetic Analyzer. Cycle Sequencing was performed with an initial 1 min denaturation at 96 °C, followed by 25 amplification cycles (10 s at 96 °C, 5 s at 50 °C, 75 s 60 °C). Sequences were aligned and compared to the reference sequences (NG_008191.1 and NC_000006.12) using the Sequencer 5.0 software (Gene Codes Corporation, Ann Arbor, USA).

Pre-designed TaqMan® SNP genotyping assays (Applied Biosystems, Foster City, CA, USA) were obtained for rs4420638, rs1061170, and rs13278062. Custom designed TaqMan® assays (Assay-by-Design, Applied Biosystems) were obtained for all other SNPs. Allelic discrimination was performed on a 7500 Fast Real-Time PCR System (Applied Biosystems) according to the manufacturer’s protocol.

### Enterotyping

The samples were clustered based on relative genus abundances using Jensen–Shannon distance and the partitioning around medoids (PAM) algorithm. The optimal number of clusters was calculated by the Calinski–Harabasz (CH) index. Between-class-analysis (BCA), a particular case of PCA, was used to visualize the taxonomic drivers of the clusters^19^.

### Statistical analysis

Demographics were compared among groups applying either Welch’s *t*-test (for age and BMI) or Fisher’s exact test (for sex and smoking, Table 1). In order to identify differences in microbial abundances, the Wilcoxon rank sum test was performed. To detect an association between enterotypes and the disease status, Fisher’s exact test was applied using GraphPad Prism version 7.04 (GraphPad software Inc.). *p*-values < 0.05 were considered to be significant.

The association analyses between AMD and the SNPs were conducted using Fisher’s exact test implemented in PLINK v.1.07^53^. Multiple testing was corrected using Benjamini–Hochberg procedure. For subsequent analysis, genotypes at each locus of each individual are coded as follows: 0 for major allele homozygous, 1 for heterozygous, and 2 for minor allele homozygous.

R software (version 3.5.1) was used to perform other analyses. To provide global analysis of microbial and pathway abundances between groups by PCA, the R package *ade4*^54^ was applied. PCA was done using scaled values on relative abundances of microbial species identified by MetaPhlAn2 and of pathways identified by HUMAnN2. A visualization of the individual samples grouped by case and control is provided in Figs. 1c, 3a, 5c, and 6a. To provide a *p*-value for separation, permutation multivariate analysis of variance (PERMANOVA) using the R package *vegan*^55^ was assessed with 1000 permutations. Associations of microbial and functional abundances with clinical metadata were analyzed using multivariate association by linear models (MaAsLin)^56^ R package. Significant association was considered below a *q*-value threshold of 0.20 after adjusting for false discovery rate (FDR; Benjamini–Hochberg). For clinical metadata, the R package *corrplot* was used for visualization of the data in a correlation matrix (Fig. 4b). The R package *sda* provided the functionality for high-dimensional linear discriminant analysis with feature selection. A species-based classifier using the relative species abundance profile from MetaPhlAn was trained using Stein-type shrinkage estimators and features are ranked according to correlation-adjusted *t* (cat) scores to provide a set of candidate biomarkers (Fig. 4a; Supplementary Fig. 1). The minimum error was calculated by 10-fold cross-validation by the “rfcv” function of the *randomForest* package with sequentially reduced numbers of features. The optimal number of features was selected by cross-validation. The model was tested on the testing set using this set of features and a receiver operating characteristic (ROC) curve within the *pROC* package in R.

### Mice

Adult (6–8 weeks of age) C3^−/−^ mice on a C57BL/6 background (B6;129S4-C3^tm1Crr^/J, *n* = 16) and C57BL/6 mice as controls (*n* = 16), both from Jackson Laboratory (Bar Harbor, ME, USA), were used for this study. Mice were housed in groups of four animals of the same gender under temperature and humidity-controlled conditions in individually ventilated cages giving sterile food and water ad libitum and exposed to 12:12 h light:dark cycles. One and 4 weeks after arrival of the mice, feces were collected and immediately frozen at −20 °C. At the end of the study, mice were killed by carbon dioxide (CO_2_) inhalation. Metagenomic DNA was isolated and sequenced as described above. To exclude low-quality reads and reads mapping to murine DNA, the resulting 150 bp paired-end reads were quality filtered with Trimmomatic v.0.32^48^ and mapped to the mouse reference genome GRCm38 using Bowtie2 v.2.3.0^49^. The high-quality non-murine reads were taxonomically profiled using MetaPhlAn2 and functionally profiled using HUMAnN2. Associations of microbial and pathway abundances with genetics (C3^−/−^ versus wildtype mice) and demographic parameters (group, sex, and week at collection) were identified by MaAsLin (for details see above). All animal studies were conducted at the University of Bern and approved by the local Animal Ethics Committee (Veterinärdienst des Kantons Bern/BE99-16).

### Reporting summary

Further information on research design is available in the Nature Research Reporting Summary linked to this article.

## Supplementary information

Supplementary Information

Reporting Summary

## Data Availability

The data sets supporting the conclusions of this article are available in the European Nucleotide Archive under accession numbers PRJEB24557, PRJEB35615, and PRJEB38145.

## Code availability

Code for taxonomical analysis using MetaPhlAn2 v.2.6.0 with description for installation is freely available at https://github.com/biobakery/MetaPhlAn. Code for functional annotation using HUMAnN2 v.0.11.05 with description for installation is freely available at https://github.com/biobakery/humann. Code for association studies using MaAsLin2 is available at https://github.com/biobakery/Maaslin2. R codes used for PCA by the package *ade4* is described at http://www.sthda.com/english/articles/31-principal-component-methods-in-r-practical-guide/119-pca-in-r-using-ade4-quick-scripts/, machine learning approaches by *sda* are described at https://rdrr.io/cran/sda/man/sda.html. More detailed informations are found in the “Methods” section and available upon request by the corresponding author.
