# Supplementary material for: Associations of the intestinal microbiome with the complement system in neovascular age-related macular degeneration
Source: NPJ Genom Med. 2020 Sep 1;5:34. doi: 10.1038/s41525-020-00141-0 (PMC7463023; doi:10.1038/s41525-020-00141-0)
Supplement: Supplementary file 1 — Supplementary Information [file 41525_2020_141_MOESM1_ESM.pdf]

# Supplementary Information

### Supplementary Table 1. Single nucleotide polymorphisms in AMD

| SNP | Gene | SNP rs number | Assay ID |
|---|---|---|---|
| g.13539G>C | C2 | rs1883025 | C_29531804_10 |
| c.304C>G | C3 | rs4420638 | C_26330755_10 |
| g.56959412C>A | CETP | rs10490924 | C_27513218_10 |
| c.26T>A | CFB1 | rs4151667 | Sanger sequencing |
| c.94C>T | CFB2 | rs12614 | Sanger sequencing |
| c.95G>C/G>T | CFB3 | rs641153 | Sanger sequencing |
| c.184G>A | CFH1 | rs800292 | C_2530382_10 |
| c.2237-543G>A | CFH2 | rs1410996 | C_2530294_10 |
| c.1204C>T | CFH3 | rs1061170 | AHZAGDA |
| g.109737911T>C | CFI | rs10033900 | C_34681305_10 |
| c.-129+1707T>C | COL8A1 | rs13095226 | C_26159211_10 |
| c.-625G>A | HTRA1 | rs11200638 | C_31018186_10 |
| g.58386313C>T | LIPC | rs10468017 | C_29910029_10 |
| c.1-113547 A>C | TIMP3 | rs9621532 | C_30611370_10 |
| c.-397C>A | TNFRSF10A | rs13278062 | AH1SCPQ |
| c.607-1400T>C | VEGF A | rs1413711 | AH21AVY |

**Supplementary Figure 1. Top 5 ranked biomarkers for C3 deficiency**

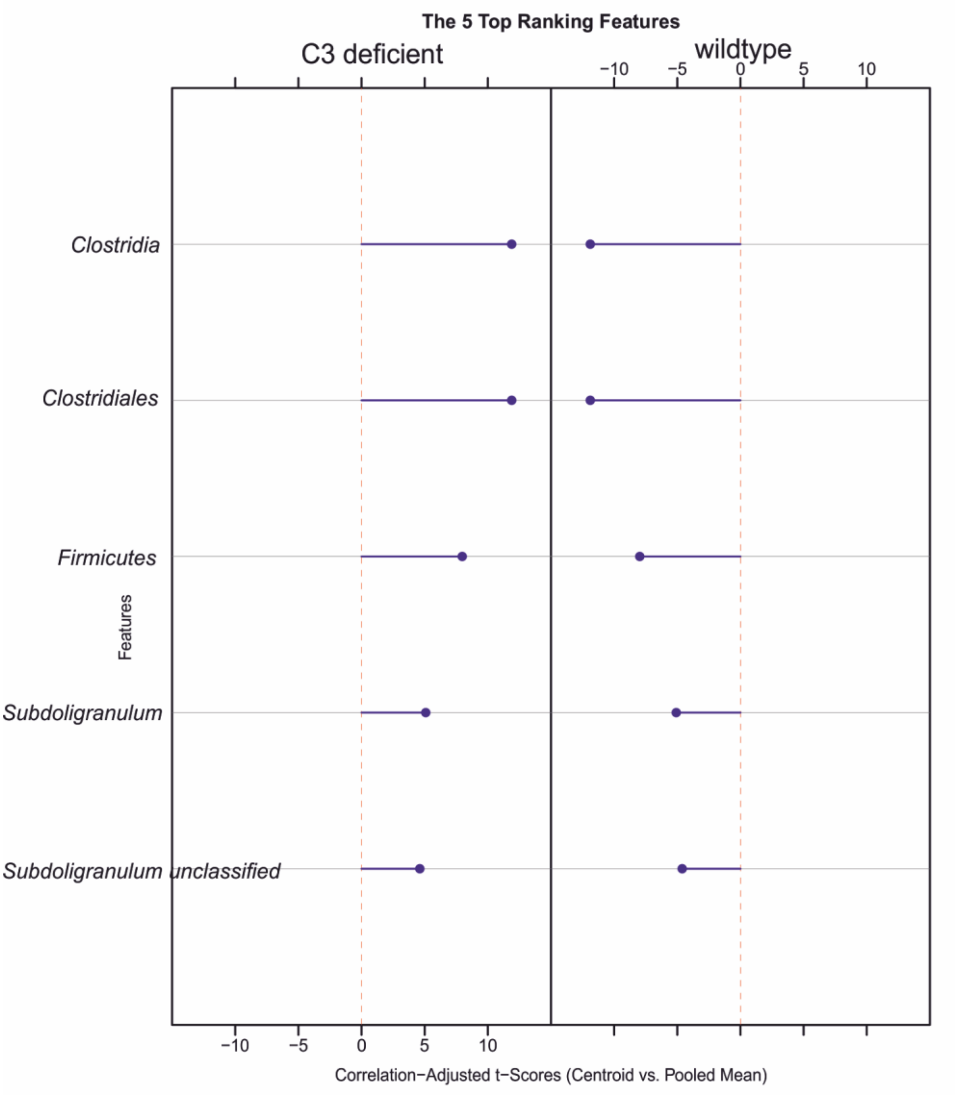


List of the top 5 ranked biomarkers for C3 deficiency. Ranking was performed on correlation adjusted *t* (cat) scores. The length and direction of the blue bars indicated the influence of a given biomarker on the discriminative power of the prediction model. The class *Clostridia* had the highest potential for the separation of C3 deficient mice and wildtypes with a positive cat score indicating an over-representation in C3 deficient mice.
