# Supplementary material for: Associations of the intestinal microbiome with the complement system in neovascular age-related macular degeneration
Source: NPJ Genom Med. 2020 Sep 1;5:34. doi: 10.1038/s41525-020-00141-0 (PMC7463023; doi:10.1038/s41525-020-00141-0)
Supplement: Supplementary file 2 — Reporting Summary [file 41525_2020_141_MOESM2_ESM.pdf]

nature research

Corresponding author(s): Denise C. Zysset-Burri

Reporting Summary

Nature Research wishes to improve the reproducibility of the work that we publish. This form provides structure for consistency and transparency in reporting. For further information on Nature Research policies, see Authors & Referees and the Editorial Policy Checklist.

Statistical parameters

When statistical analyses are reported, confirm that the following items are present in the relevant location (e.g. figure legend, table legend, main text, or Methods section).

n/a	Confirmed	
☐	☒	The exact sample size (n) for each experimental group/condition, given as a discrete number and unit of measurement
☐	☒	An indication of whether measurements were taken from distinct samples or whether the same sample was measured repeatedly
☐	☒	The statistical test(s) used AND whether they are one- or two-sided<br>*Only common tests should be described solely by name; describe more complex techniques in the Methods section.*
☒	☐	A description of all covariates tested
☐	☒	A description of any assumptions or corrections, such as tests of normality and adjustment for multiple comparisons
☐	☒	A full description of the statistics including central tendency (e.g. means) or other basic estimates (e.g. regression coefficient) AND variation (e.g. standard deviation) or associated estimates of uncertainty (e.g. confidence intervals)
☐	☒	For null hypothesis testing, the test statistic (e.g. F, t, r) with confidence intervals, effect sizes, degrees of freedom and P value noted<br>*Give P values as exact values whenever suitable.*
☒	☐	For Bayesian analysis, information on the choice of priors and Markov chain Monte Carlo settings
☐	☒	For hierarchical and complex designs, identification of the appropriate level for tests and full reporting of outcomes
☒	☐	Estimates of effect sizes (e.g. Cohen's d, Pearson's r), indicating how they were calculated
☐	☒	Clearly defined error bars<br>*State explicitly what error bars represent (e.g. SD, SE, CI)*

Our web collection on statistics for biologists may be useful.

Software and code

Policy information about availability of computer code

Data collection	For data collection no software was used.
Data analysis	Code for taxonomical analysis using MetaPhlAn2 v.2.6.0 with description for installation is freely available at https://github.com/biobakery/MetaPhlAn. Code for functional annotation using HUMAnN2 v.0.11.05 with description for installation is freely available at https://github.com/biobakery/humann. Code for association studies using MaAsLin2 is available at https://github.com/biobakery/Maaslin2. R codes used for PCA by the package ade4 is described at http://www.sthda.com/english/articles/31-principal-component-methods-in-r-practical-guide/119-pca-in-r-using-ade4-quick-scripts/, machine learning approaches by sda are described at https://rdrr.io/cran/sda/man/sda.html. More detailed information are found in the Methods section and available upon request by the corresponding author.

For manuscripts utilizing custom algorithms or software that are central to the research but not yet described in published literature, software must be made available to editors/reviewers upon request. We strongly encourage code deposition in a community repository (e.g. GitHub). See the Nature Research guidelines for submitting code & software for further information.

April 2018

Data

Policy information about availability of data

All manuscripts must include a data availability statement. This statement should provide the following information, where applicable:

- Accession codes, unique identifiers, or web links for publicly available datasets
- A list of figures that have associated raw data
- A description of any restrictions on data availability

The datasets supporting the conclusions of this article are available in the European Nucleotide Archive under accession numbers PRJEB24557, PRJEB35615 and PRJEB38145

Field-specific reporting

Please select the best fit for your research. If you are not sure, read the appropriate sections before making your selection.

☒ Life sciences ☐ Behavioural & social sciences ☐ Ecological, evolutionary & environmental sciences

For a reference copy of the document with all sections, see nature.com/authors/policies/ReportingSummary-flat.pdf

Life sciences study design

All studies must disclose on these points even when the disclosure is negative.

Sample size	The sample size was determined based on a power analysis with a power of 80% and an alpha of 0.05.
Data exclusions	Exclusion criteria were chronic inflammatory or gastrointestinal diseases (including previous surgery in the gastrointestinal tract) and use of systemic antibiotics within the last three months.
Replication	The replication of whole genome sequencing data is described else where.
Randomization	Since this is a case-control study without medication, a randomization is not relevant.
Blinding	Since this is a case-control study without medication, a blinding is not relevant.

Reporting for specific materials, systems and methods

Materials & experimental systems

n/a	Involved in the study
☒	☐ Unique biological materials
☒	☐ Antibodies
☒	☐ Eukaryotic cell lines
☒	☐ Palaeontology
☐	☒ Animals and other organisms
☐	☒ Human research participants

Methods

n/a	Involved in the study
☒	☐ ChIP-seq
☒	☐ Flow cytometry
☒	☐ MRI-based neuroimaging

Animals and other organisms

Policy information about studies involving animals; ARRIVE guidelines recommended for reporting animal research

Laboratory animals	Adult (6-8 weeks of age) C3-/- mice on a C57BL/6 background (B6;129S4-C3tm1Crr /J, n = 16, 8 females, 8 males) and C57BL/6 mice as controls (n = 16, 8 females, 8 males), both from Jackson Laboratory (Bar Harbor, ME, USA), were used.
Wild animals	The study does not involve wild animals.
Field-collected samples	The study does not involve samples collected from the filed.

Human research participants

Policy information about studies involving human research participants

Population characteristics	All participants (n = 115) were Caucasian. Patients (n = 57, 21 males, 36 females) had clinically confirmed active neovascular AMD and were 50 years of age or older, and the control group (n = 58, 25 males, 33 females) was selected to represent an age- and sex-matched group with no signs of AMD.
Recruitment	Participants were recruited from the Department of Ophthalmology of the University Hospital Bern (Inselspital), Switzerland.
